# Supplementary material for: A Global Screen for Assembly State Changes of the Mitotic Proteome by SEC-SWATH-MS
Source: Cell Syst. 2020 Feb 26;10(2):133–155.e6. doi: 10.1016/j.cels.2020.01.001 (PMC7042714; doi:10.1016/j.cels.2020.01.001)

Q9NZI8 | IF2B1_HUMAN | IGF2BP1 CRDBP VICKZ1 ZBP1
Monomer MW [kDa]: 63.481 Monomer expected elution fraction: 44

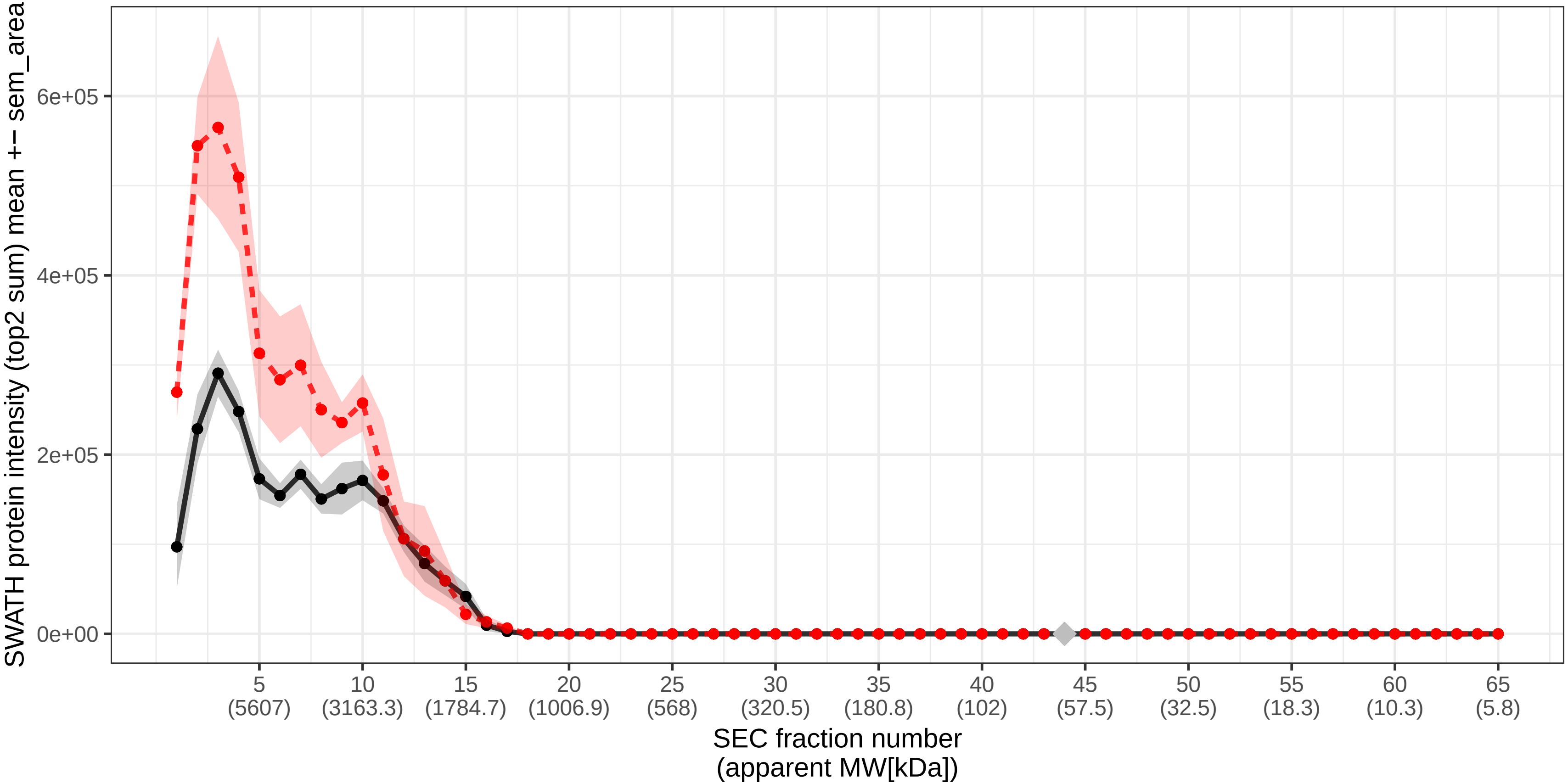

Supplement: Data S1. SEC-SWATH-MS Protein Chromatograms, Related to Figure 1 [file mmc6.zip › SECchrom_Q9NZI8_IF2B1_HUMAN_IGF2BP1_CRDBP_VICKZ1_ZBP1.pdf]
